# Pennsylvania College of Dental Surgery

**Published:** 1869-04

**Authors:** 


### ARTICLE VII.

### *Pennsylvania College of Dental Surgery.*

The Thirteenth Annual Commencement of this College was held at the Musical Fund Hall, Philadelphia, February 27th The Valedictory Address was delivered by Professor George T. Barker. The degree of "Doctor of Dental Surgery" was conferred upon the following regular graduates, twenty-four in number:

G. W. Adams, *Pennsylvania;* Wm. N. Baumgartner, *Maryland;* H. D. Bennett, *Illinois;* A. L. Betancourt, *Cuba;* Jacob E. Brecht, *Pennsylvania;* B. Climenson, *Pennsylvania;* J. P. Crowell, *California;* John W. Crymes, *South Carolina;* J. H. Downes, *Pennsylvania;* R. R. Freeman, *Tennessee;* H. Hirschfeld, *Prussia;* S. H. Linn, *Delaware;* Lorenzo J. Martin, *Cuba;* Thomas J. Mitchell, *North Carolina;* T. S. Muridge, *Washington*

*Territory* ; J. W. Moore, *Pennsylvania* ; A. E. Peyrellade, *Cuba* ; J. E. Register, *Maryland* ; W. H. Roop, *Pennsylvania* ; C. Rohland, *Pennsylvania* ; B. L. Taylor, *Minnesota* ; S. B Tizzard, *Ohio* ; F. R. Thomas, *Pennsylvania* ; D. Van Buskirk, *Pennsylvania.*

